# Echocardiography in the Diagnosis of Cardiomyopathies: Current Status and Future Directions 

**DOI:** 10.31083/j.rcm2308280

**Published:** 2022-08-10

**Authors:** Livia Trasca, Mihaela Roxana Popescu, Andreea Catarina Popescu, Serban Mihai Balanescu

**Affiliations:** ^1^Cardiothoracic Medicine Department, “Carol Davila'' University of Medicine and Pharmacy, 020021 Bucharest, Romania; ^2^Department of Cardiology, Elias Emergency University Hospital, 11461 Bucharest, Romania

**Keywords:** restrictive cardiomyopathy, dilated cardiomyopathy, speckle tracking, arrhythmogenic right ventricle cardiomyopathy, ventricular non-compaction, stress cardiomyopathy, Takotsubo syndrome, deep learning

## Abstract

Cardiomyopathies are a challenging pathology and echocardiography is essential for diagnosis and prognosis. The most frequent cardiomyopathies are the dilated cardiomyopathy (DCM) and the hypertrophic cardiomyopathy (HCM), followed by the less frequent restrictive (RCM) and arrhythmogenic right ventricle cardiomyopathies (ARVC). Echocardiography can identify diagnostic features, and guide further testing for a definitive diagnosis. Echographic parameters are involved in risk score computing and prognosis assessment. While the most prevalent hallmark of HCM is the asymmetric left ventricular hypertrophy and systolic anterior motion of the mitral valve with the obstructive phenotype, DCM shows dilated left ventricle with different degrees of systolic dysfunction, and RCM is usually characterized by undilated ventricles associated with atrial enlargement. The aim of this review is to display and compare the most frequent cardiomyopathies encountered in clinical practice and highlight their most characteristic features in a useful way for the practicing clinician.

## 1. Introduction

Echocardiography is the main diagnosis and monitoring technique for various types of heart diseases. It has a favorable cost-efficiency ratio, low risk, allows follow-up, and offers increasingly more accurate information due to continuous development in terms of technique, imaging methods, and pharmacology-associated assessment. Moreover, ultrasonography machines have undergone significant development in recent years Thus echocardiography continues to present several advantages over others imaging diagnostic techniques [1].

Cardiomyopathies represent a variety of myocardial disorders with damage to the heart muscle as a common feature. There are various functional and structural phenotypes with or without hereditary transmission [1, 2]. Heart muscle damage is associated with the primary impairment, therefore structural and functional changes that appear secondary to ischemic, congenital, or valvular heart disease are not considered to be part of the “cardiomyopathy” spectrum. Approved by the World Heart Federation in 2013, the most complete classification of cardiomyopathy is MOGE(S) [2]. Each one of the four capital letters represents a framing feature of cardiomyopathies referring to morphologic and functional characterization (M), the presence of other organs involvement (O), the pattern of hereditary transmission or in other words the genetic status (G), etiology (E) and stage of the evolution (S) [2]. From a morphological point of view five types of cardiomyopathies are described: dilated, hypertrophic, restrictive, arrhythmogenic right ventricle cardiomyopathy and unclassified cardiomyopathy including ventricular non-compaction and stress (Takotsubo) cardiomyopathy [3].

In the majority of cases, the diagnosis of cardiomyopathy is established after the onset of symptoms, which can be related to heart failure, and atrial or ventricular arrhythmias. Therefore, the first imaging investigation used to assess a patient with suspicion of cardiomyopathy is echocardiography, which is widely available, usually reliable and reproducible, cost-effective, and risk-free [4]. From the estimation of left and right heart filling pressures to the evaluation of the systolic and diastolic function of the left ventricle through several complex techniques, the cardiac ultrasonography can provide information related to the diagnosis, staging, and response to treatment or prognostic parameters in different cardiomyopathies [1, 5]. Bi-dimensional and three-dimensional echocardiography may be used and provide information for complex characterization of the heart in cardiomyopathies from basic parameters, like anatomic measurements or estimation of the systolic and diastolic function of the ventricles, to a more particular assessment of the muscle tissue through techniques like speckle tracking and tissue Doppler imaging [1].

This review aims to summarize the different echocardiographic characteristics observed in the above-mentioned cardiomyopathies and highlight the specific use of echocardiographic techniques in particular situations.

## 2. Dilated Cardiomyopathy

Dilated cardiomyopathy (DCM) is defined by dilatation associated with impaired contraction of one or both ventricles. The definition of cardiomyopathy refers to primary myocardial dysfunction of unknown etiology, with autosomal or sex-linked, dominant or recessive genetic inheritance, or as an acquired disorder with infectious (post myocarditis) or toxic origin. Both American and European Societies’ classification systems do not consider the ischemic cardiac disease, with criteria for dilation and impaired systolic function of the left ventricle (LV), as a cause of cardiomyopathy. This association between ischemia and myocardial dilatation and dysfunction is described as a separate disease [1, 2, 6].

Dilated cardiac chambers due to ischemic heart disease are associated with specific echocardiographic anomalies, like regional wall contractility disturbances or regional remodeling. However, this kind of limited wall segment motion abnormality can also be found in idiopathic DCM. For example, other diseases that can be characterized by regional wall motion abnormalities include sarcoidosis or tuberculosis. Ischemic cardiomyopathy may also present areas of endocardial brightening or scarring in infarcted areas [7].

### 2.1 2D Echocardiography

**Fig. 1.**
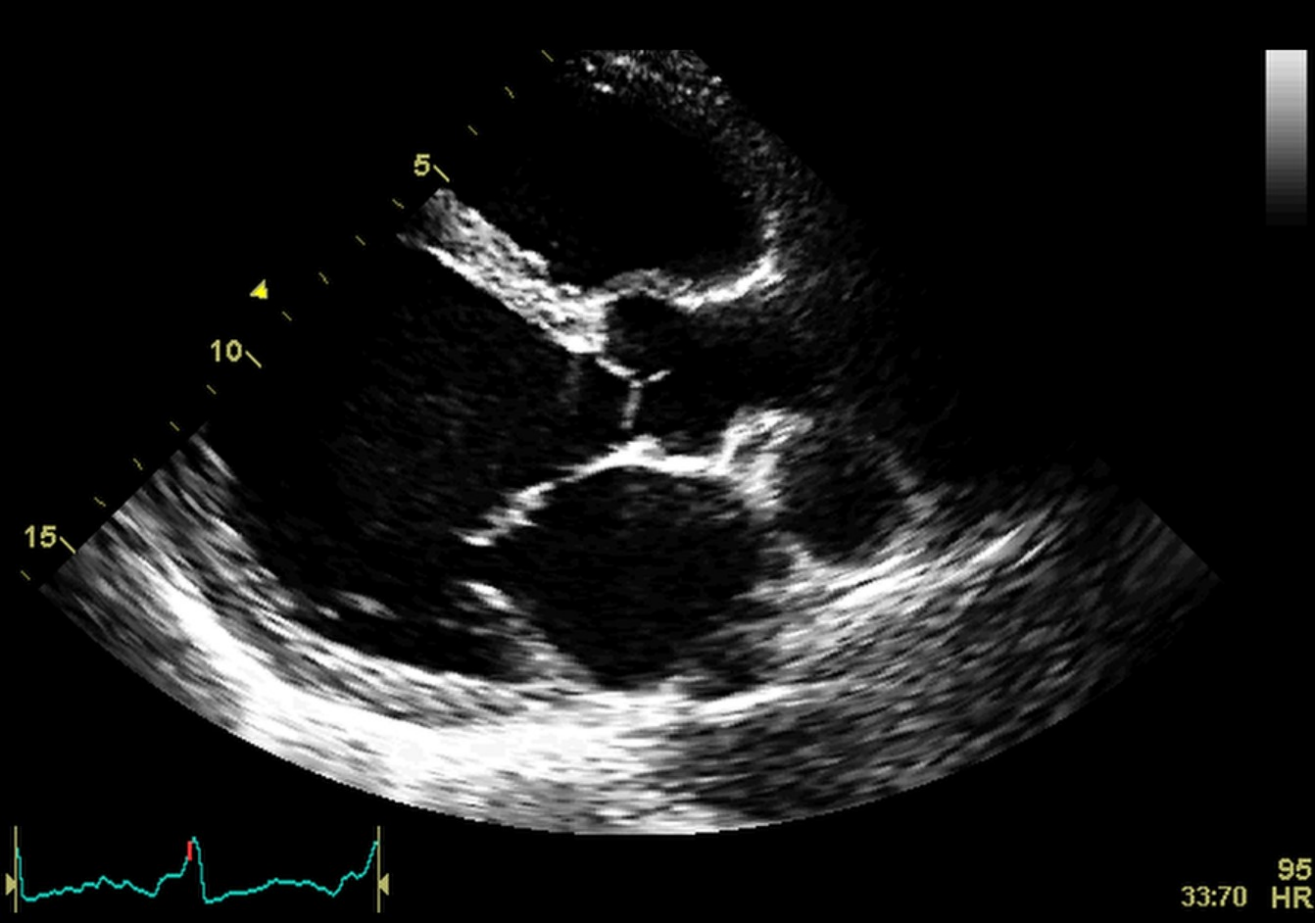
**Echocardiographic aspect of DCM**. Parasternal long-axis view showing a dilated left atrium and left ventricle in diastole. An online video of this patient with idiopathic dilated cardiomyopathy is available (Appendix Video 1).

The initial imaging evaluation comprises of two main 2D echocardiography parameters: left ventricle ejection fraction (LVEF) less than 40% or fractional shortening less than 25% [6]. However, the comprehensive diagnosis of DCM should consider aspects of the clinical presentation, patient examination and other test results. Dilatation of the LV, especially end-diastolic and end-systolic transverse diameter enlargement (spherical remodeling), associated with reduced wall thickness, is easily recognized with 2D echocardiography in parasternal long-axis view (See Fig. 1 and Appendix Video 1).

**Fig. 2.**
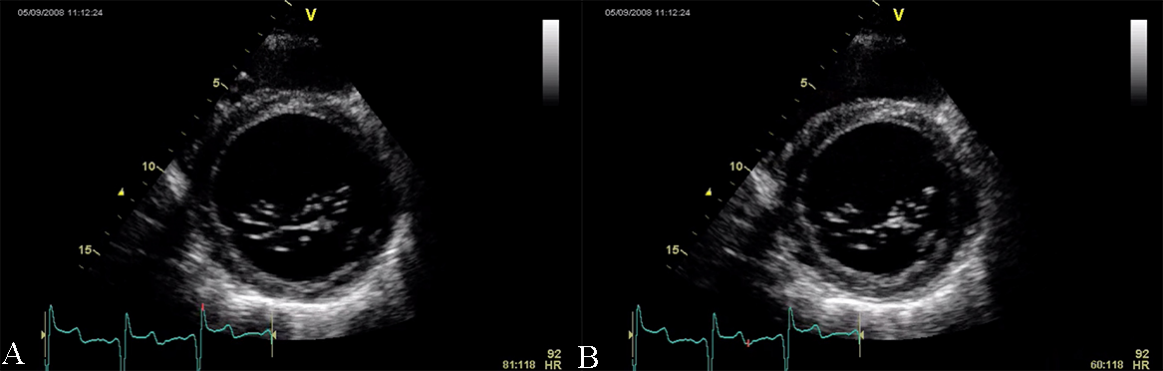
**Echocardiographic aspect of DCM in a patient with idiopathic dilated cardiomyopathy**. (A) Parasternal short axis at the level of the mitral valve, diastolic frame showing a dilated left ventricle. (B) Parasternal short axis at the level of the mitral valve, systolic frame showing the small difference between the end-diastolic and end-systolic diameter of the left ventricle due to reduced contractility.

Cardiac chamber enlargement is often present, but linear volumetric estimation is no longer recommended. According to the American Society of Echocardiography quantitative parameters should be obtained for an accurate assessment. Thus, DCM is defined by an LV end-diastolic volume index above 100 mL/$m^{2}$ [4]. The upper normal volume is 74 mL/$m^{2}$ in men and 61 mL/$m^{2}$ in women [4]. The end-systolic volume of the LV is a defining feature, but also a prognostic parameter, an index greater than 35 mL/$m^{2}$ is correlated with an unfavorable outcome [3, 4]. Another classic parameter is the LVEF, less than 40% and is specific to heart failure with reduced ejection fraction (HFrEF) (see Fig. 2). It is widely accepted that patient outcome is inversely related to the left ventricle ejection fraction. The hallmark of DCM is left ventricular cavity dilation, which may be associated with other cardiac chamber enlargements. Although the myocardial walls may be either of normal or reduced thickness, the total left ventricular mass is increased because of the overall increase in LV size. However, parameters related to the LV systolic function, such as fractional shortening, ejection fraction, stroke volume, and cardiac output are typically reduced. The normal range of the stroke volume is between 50 and 100 mL/beat. Although the stroke volume is reduced in some cases, LV cavity dilation may initially serve to compensate by restoring the stroke volume, thus it is recommended to guide the diagnosis by measuring the LV end-systolic volume index (ESVI) because it provides important information in several clinical settings.

Left atrial volume index (LAVi) is estimated from 2D echocardiography 4 chamber-view. A value above 50 mL/$m^{2}$ is a characteristic echocardiographic aspect of DCM [8]. This is due especially to the diastolic disfunction associated with different grades of mitral regurgitation. Data suggest that minimal atrial volume assessed at ventricular end-diastole has a better predictive value of outcomes than maximal left atrial volume measured at ventricular end-systole [9].

The evaluation of the anatomy and function of the right ventricle (RV) is mandatory, given the fact that the prognosis is considerably worse when there is RV dilatation and/or RV systolic dysfunction. RV systolic power is defined by the tricuspid annular plane systolic excursion (TAPSE) in 2D apical 4-chamber view, ejection fraction and fractional area change. For TAPSE, a reported value of 14 mm, is associated with poor prognostic in patients with DCM [3, 4]. RV dysfunction is not essential for the diagnosis of DCM but when present, it is an adverse prognostic marker.

### 2.2 Doppler Echocardiography

**Fig. 3.**
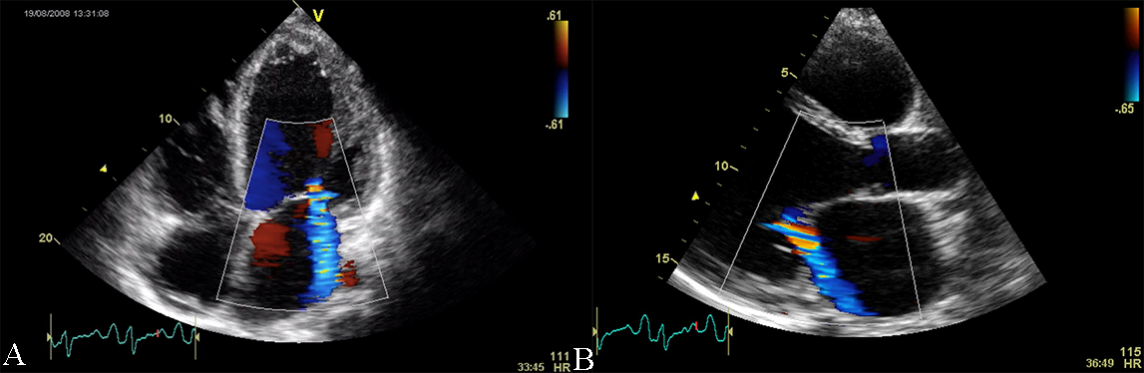
**Echocardiographic aspect of DCM in a patient with idiopathic dilated cardiomyopathy**. (A) Apical 4 chamber view, showing dilated left ventricle, dilated left atrium, and significant secondary mitral regurgitation flow with Coanda effect. (B) Parasternal long-axis view, showing dilated left ventricle, dilated left atrium, and significant secondary mitral regurgitation flow with Coanda effect. An online video of this patient with idiopathic dilated cardiomyopathy is available (Appendix Video 2 and 3).

Color and spectral Doppler echocardiography is of limited use in the diagnosis of DCM, except for atrioventricular valve incompetence. One constant feature is the presence of mitral regurgitation, secondary to the annulus dilation with abnormal leaflet tethering. Mitral regurgitation can present with different degrees of severity and is an additional prognostic factor (see Fig. 3 and Appendix Video 2 and 3). The pathophysiology of mitral regurgitation in DCM is usually due to abnormal leaflet tethering mandated by the change in LV shape from ellipsoid to spherical. As cardiomyopathy progresses, the point of leaflet coaptation migrates from its normal basal location to a spot deeper in the LV cavity this leading to also to an apposition defect with the generation of an eccentric regurgitant jet. Moreover, DCM patients present with an abnormally low diastolic function [5]. Restrictive or pseudo-normal diastolic inflow patterns impact the patients with DCM, with an associated poor prognosis [10, 11]. The assessment of the pulmonary vein flow signal as an adjunct to the mitral inflow pattern is very important in the echographic evaluation of DCM. Abnormal pulmonary venous systolic flow patterns have been shown to correlate with the future development of pulmonary hypertension. Loss of the systolic dominant flow pattern suggests elevated filling pressure and the difference between the pulmonary venous flow reversal duration and the mitral inflow duration during atrial contraction greater than 30 ms predicts mortality and hospitalization [9]. Studies evaluating diastolic compliance with Doppler echocardiography have shown also an altered mitral diastolic flow with a short deceleration time ≤115 msec, and an E/A ratio >1 is a powerful independent predictor of mortality or the need for transplantation among patients with heart failure [9].

### 2.3 Tissue Doppler Imaging (TDI)

TDI is a technique used to characterize diastolic dysfunction but also gives important information about the global and regional systolic function of the myocardium. By TDI can be assessed displacement velocities of small samples of the tissue, values which are directly proportional to the contractility force of the region of interest but it is also correlated with the global systolic function of the LV or RV. The mean normal value of the velocity contraction (S wave) for the septal annulus is 8.1 ± 1.5 (6.0–10.9) and for the lateral mitral annulus is 10.2 ± 2.4 (6.7–14.6) for a 35 to 75 years old study population [12]. Myocardial mitral annular or basal segmental (Sm) systolic and early diastolic (Ea or Em) velocities have been shown to predict mortality or cardiovascular events. In DCM the myocardial systolic velocity displacement is under the normal values. Several studies are comparing the S wave value among different age groups with no significant differences. Related to the diastolic dysfunction, Doppler tissue imaging exhibits patterns associated with elevated LV filling pressures. Mitral annular early diastolic velocity ratio (E/E TDI—annular or segmental) is a strong prognostic parameter, especially when E/E TDI is ≥15. Moreover, systolic intraventricular dyssynchrony measured by segmental analysis of myocardial velocities is another independent predictor of adverse clinical outcomes in heart failure subjects, even when the QRS duration is normal [12].

### 2.4 Strain Echocardiography

Speckle tracking is also useful in the evaluation of DCM. Left ventricle global longitudinal strain (GLS) is more sensitive compared to the ejection fraction in the detection of impaired systolic function. Moreover, the GLS is a prognostic parameter for the left ventricle reverse remodeling. A higher GLS is associated with a better reverse remodeling, even with a similar left ventricle ejection fraction. A GLS value more negative than –10%, which is reported as a cut-off, is related to better reverse remodeling and consequently with better long-term prognosis [13].

### 2.5 Echo Features in DCM Therapy

Some cases of DCM with severe systolic dysfunction (left ventricular ejection fraction ≤35%) and wide QRS complex remain symptomatic, with chronic heart failure (NYHA class II–IV) despite optimal medical treatment. This subgroup of patients may benefit from cardiac resynchronization therapy (CRT) [14]. Patients who are CRT candidates may be evaluated by echocardiography to assess LV systolic and regional function and also cardiac dyssynchrony [15, 16]. Several echocardiographic modalities for dyssynchrony assessment are employed, such as conventional M-mode and pulsed-wave Doppler techniques [15, 16, 17]. While individual studies showed strong promise for the use of echo-derived mechanical dyssynchrony in determining response to CRT [15, 16, 17], multicenter studies failed to demonstrate prognostic benefit, hence echo is not currently used to assess mechanical dyssynchrony to determine CRT response [18].

Interventricular dyssynchrony is determined by the temporal phase shift between the contraction of the two ventricles. There are several useful parameters to define dyssynchrony at the level and they can be obtained by conventional pulsed-wave Doppler or Tissue Doppler imaging. A difference higher than 40 ms between left ventricular and right ventricular pre-ejection time (measured by pulsed-wave Doppler), and also a delay greater than 56 ms between the onset of systolic motion in the basal right ventricular free wall versus the most delayed basal LV segment (measured by tissue Doppler) are the most used parameters for defining the interventricular dyssynchrony, but they also have limited value for predicting CRT response.

Intraventricular dyssynchrony defined as inhomogeneity in myocardium contractility can be evaluated by conventional echocardiography, tissue velocity measurements, and deformation imaging. The temporal difference between septal to posterior wall contraction can be assessed by M-mode echocardiography, from a parasternal short-axis view at the papillary muscle level, by measuring the delay in systolic thickening of the myocardium [17, 19]. It is calculated as the interval between the maximal posterior displacement of the septum and the maximal displacement of the left posterior wall. Another parameter used for establishing intraventricular dyssynchrony is the value of the pre-ejection time, measured from QRS onset to aortic flow onset. The recognized cut-off values are septal to posterior wall motion delay above 130 ms and left ventricular pre-ejection time higher than 140 ms [8, 20]. There is no demonstrated predictive role for these parameters. In contrast to the described parameters related to the timing of myocardial velocity peaks, myocardial deformation parameters (strain, strain rate) may help in distinguishing active contraction from passive one caused by tethering of adjacent myocardial regions [8, 14, 15, 21]. These parameters may also be used during follow-up for highlighting the reverse remodeling process, and some of them as prognostic features for patients with DCM and LV systolic dysfunction [14, 15, 17, 20].

Ventricular remodeling describes structural changes in the left ventricle in response to chronic alterations in loading conditions. Current therapeutic strategies for systolic heart failure aim to slow or halt the remodeling process. Reverse remodeling refers to a concept, where progressive LV dilatation and deterioration in contractile function are not simply arrested, but partially reversed is defined as a process characterized by a reduction in LV volumes with improvement in systolic and diastolic function. Right ventricular function normalization is part of a global hemodynamic improvement induced by therapy and precedes LV reverse remodeling.

## 3. Hypertrophic Cardiomyopathy

Hypertrophic cardiomyopathy (HCM) is defined by increased regional or global LV mass. The histological characterization includes cellular disarray and fibrosis which lead mainly to diastolic dysfunction. Systolic dysfunction is related to reduced preload with hypertrophied myocardium and reduced end-diastolic LV chamber. Proper systolic dysfunction may occur late in the course of the disease, when heart dilation may ensue. Echocardiography is the main method of diagnosis, severity classification and risk estimation in HCM [3, 4]. Echocardiographic parameters such as LV wall thickness, LA size, LVOT gradient, or the presence of apical aneurysm are included in the assessment of the 5-year sudden cardiac death risk model, as part of the protocol for primary prophylaxis of sudden cardiac death, and ICD implantation decision [22]. An IVS thickness above 30 mm is recognized as one of the criteria in decision-making for ICD implantation, as recommended by the current guidelines [23]. Also, reverse septal curvature, associated with septal HCM, predict SCD and may be included in future guidelines for ICD implantation [24, 25, 26].

**Fig. 4.**
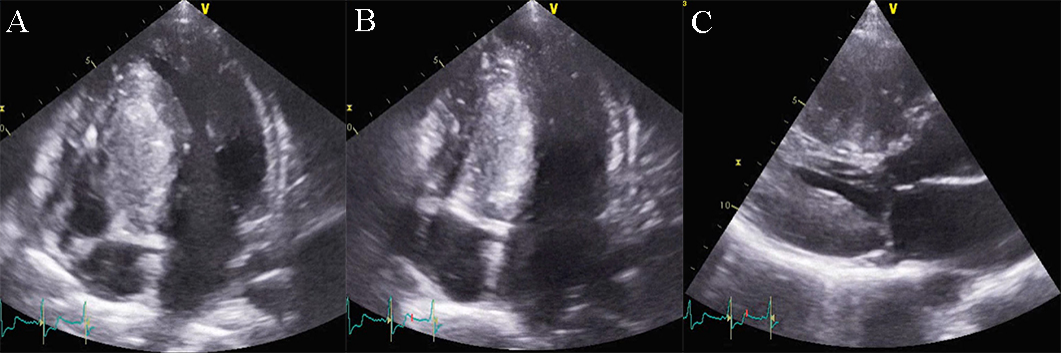
**Echocardiographic aspect of HCM**. (A) Apical 4 chamber view systolic frame, showing increased septum thickness. (B) Apical 4 chamber view diastolic frame. (C) Parasternal long-axis view- evidence of systolic anterior motion of the mitral valve. An online video of this patient with HCM is available (Appendix Video 4).

Echocardiography is the first-line diagnostic method for differential diagnosis in HCM, to identify LV hypertrophy from secondary causes. Severe hypertrophy is diagnosed when the LV mass is above 130 g/$m^{2}$ for men and >112 g/$m^{2}$ for women, a common finding in primary hypertrophy due to HCM (see Fig. 4). Moreover, the hypertrophy is usually asymmetric in inherited HCM and symmetric in secondary disease [7, 10, 27]. Displacement of the papillary muscle and chordae tendineae, long anterior mitral leaflet and systolic anterior motion (SAM) (see Fig. 4 and Appendix Video 4), accompanied by secondary LV outflow gradient and mitral regurgitation, associated with poor contractility evidenced by speckle tracking and tissue Doppler imaging, are specific criteria for HCM. These criteria are useful to help in the differential diagnosis from LV secondary hypertrophy [28].

### 3.1 2D Echocardiography

**Fig. 5.**
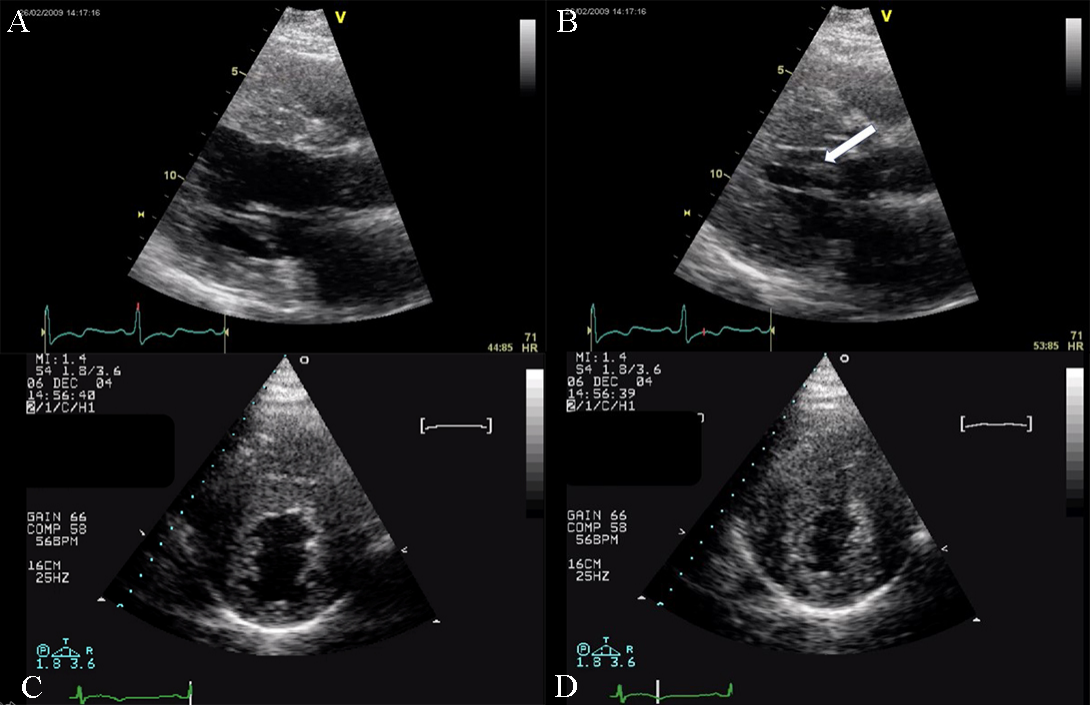
**Echocardiographic aspect of HCM**. (A) Parasternal long-axis view diastolic frame showing increased thickness of septum and inferolateral wall. (B) Parasternal long-axis view systolic frame showing (arrow) systolic anterior motion of the anterior mitral valve. (C) Short axis view diastolic frame showing significant left ventricular hypertrophy. (D) Short axis view systolic frame showing reduced left ventricular end-systolic diameter.

2D echocardiography in HCM detects a septal wall thickness higher than 14 mm as a diagnostic criterion for HCM, after excluding all the other causes of LV hypertrophy. The severity and distribution of hypertrophy in HCM are highly variable [23]. It may be concentric or localized (septal, apical, free LV wall, or right ventricle hypertrophy) with or without intraventricular obstruction (medio-ventricular, LV outflow). One pathognomonic echocardiographic feature is the asymmetric septal hypertrophy, with a ratio of septal to the posterior wall thickness of 1.5/1 [29]. The first echocardiographic criterion for the diagnosis of LV hypertrophy is the LV mass obtained by M-mode and defined by an LV mass index ≥134 g/$m^{2}$ body surface area in men and ≥110 g/$m^{2}$ in women. Current guidelines define severe hypertrophy with an LV mass above 130 g/$m^{2}$ for men and >112 g/$m^{2}$ for women [4]. An LV mass index having a value above 95 g/$m^{2}$ indicates an abnormally hypertrophied LV [5, 28, 29, 30]. In obstructive HCM, another echocardiographic specific sign is an abnormal anterior motion of the mitral valve, which tractions the anterior mitral leaflet within the LV outflow tract against the IV septum due to anterolateral papillary muscle embedded in hypertrophic myocardium (see Fig. 5). The duration of the contact between the anterior mitral valve and the LV septum in systole determines the severity of the outflow pressure gradient [30]. Depending on the predominant localization of segmental myocardial hypertrophy, different HCM phenotypes can be distinguished including asymmetric (septal) involvement which is the most common form of the disease, and other variants including apical, symmetric, midventricular, mass-like, and noncontiguous HCM. According to the localization of the hypertrophy, the dynamic obstruction may be assessed at the outflow tract or medio-ventricular. One important prognostic parameter in patients with HCM is the dimension of the left atrium, its size is associated with a higher risk for adverse events. Enlarged LAVi in HCM is associated with the severity of diastolic dysfunction. A transverse diameter larger than 48 mm and a volume above 118 ml are related to a higher risk of atrial fibrillation onset and poor survival in patients with HCM [28, 30]. Malposition of the anterolateral papillary muscle with anterior displacement may be observed in HCM, causing the outflow tract gradient. Moreover, the calcification of the mitral annulus which is a sign frequently found in HCM is related to the dynamic outflow tract obstruction [28, 30].

Echocardiography may also identify a phenotype of HCM associated with small apical aneurysms, not due to ischemia. These patients frequently present with malignant ventricular arrhythmias and resuscitated cardiac death or systemic cardioembolism [31]. Thus, the presence of apical aneurysms in the setting of HCM is associated with an ominous outcome and needs prompt implantation of an ICD [32].

### 3.2 Doppler Echocardiography

**Fig. 6.**
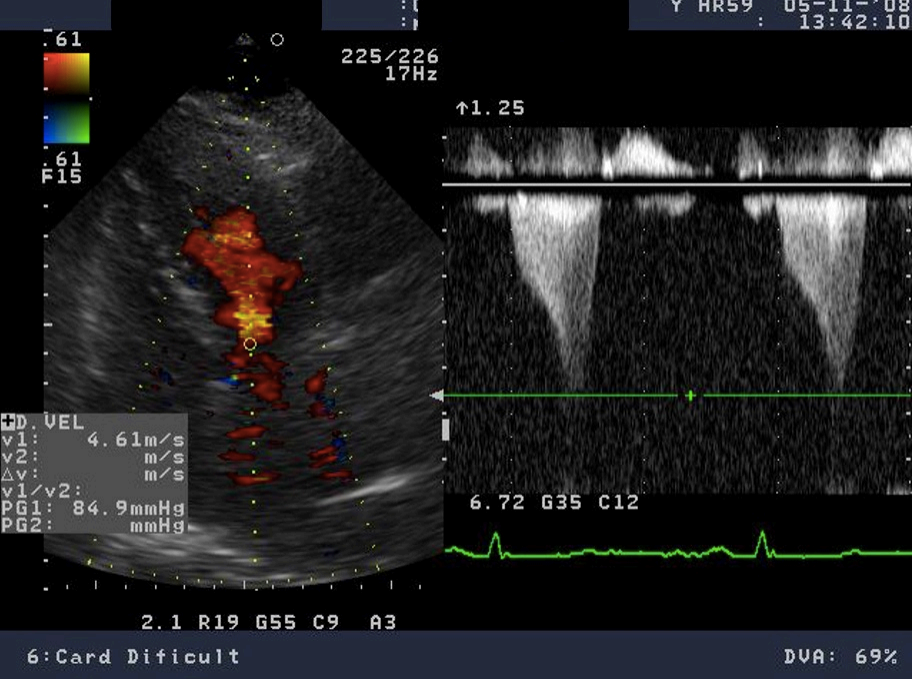
**Echocardiographic aspect of HCM**. CW Doppler in a patient with obstructive hypertrophic cardiomyopathy shows typical systolic flow with a late peaking gradient of 85 mm Hg at end-systole. The aspect of the continuous Doppler curve is typical for obstructive HCM, with an early peak and major increase of meso-telesystolic velocities when intraventricular obstruction occurs. This is most obvious in relatively bradycardic patients who display the typical “pulsus bisferiens” pattern.

Doppler echocardiography allows assessment of the intraventricular gradient and associated mitral regurgitation. One specific feature is the variability of the LV outflow gradient according to the hydration status, heart rate, the Valsalva maneuver (preload), and blood pressure (afterload). Conditions or pharmacologic interventions that increase preload and increase LV end-diastolic volume may reduce LVOT obstruction. Reduced preload and tachycardia responsible for a reduction in LV end-diastolic volume are associated with an increase in intraventricular gradient. Stress echocardiography is a method that employs different agents (exercise, dobutamine, isoproterenol, amyl nitrite) which alter LV diastolic volume, to highlight the dynamic intraventricular gradient [5, 27, 33]. The degree of mitral regurgitation severity in HCM can vary from mild to severe. The mechanism of mitral regurgitation is related to the geometry change of the valve due to the malposition of the anterolateral papillary muscle, the abnormal attachment of primary chordae tendineae and SAM. The latter is favored by the elongated leaflets of the mitral valve which are a usual feature of the mitral valve in HCM. Moreover, some studies establish a direct linear relationship between the left ventricular outflow tract area and the mitral valve area, with a positive correlation with the severity of outflow tract obstruction (see Fig. 6) [27, 33].

### 3.3 Tissue Doppler Imaging (TDI)

TDI offers important and early information about the subclinical systolic dysfunction showed by under normal values of the myocardium velocity contraction [34]. The explanation for this is the disarray among the myocytes and the increased fibrotic tissue. An early diagnosis and prognostic parameter assessing the systolic function of the LV in HCM is the longitudinal dysfunction by an S lateral value under <9 cm/s. This constitutes a criterion for differentiating pathological LV hypertrophy (HCM/hypertensive LV hypertrophy) from physiological LV hypertrophy. Moreover, a lateral mitral annular systolic velocity lower than 4 cm/s was found to have a prognostic value and independently predicted death or hospitalization for worsening heart failure [26, 35]. There are also data suggesting that systolic (Sa) and diastolic (Ea) myocardial velocities measured by TDI are decreased in subjects who have mutations causing HCM, but who do not have yet developed LV hypertrophy [26, 36].

### 3.4 Strain Echocardiography

**Fig. 7.**
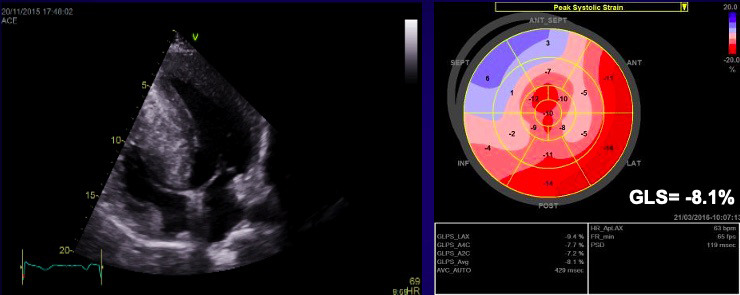
**Echocardiographic aspect of HCM**. (A) Apical 4 chamber view shows left ventricular hypertrophy. (B) The longitudinal strain bull’s eye plot derived from 2-D speckle tracking imaging shows a significantly reduced GLS (global longitudinal strain) of –8.1%, the most abnormal region is the basal septum, corresponding with the greatest wall thickness.

The most specific feature of the myocardium in HCM is the reduced contractile stress (the force per unit area) because of the cardiomyocyte disarray and interstitial fibrosis [33, 37]. A maintained ejection fraction is explained by the increased end-diastolic wall thickness that produces an augmented thickening, but the histopathology of this disease is characterized by myocardial hypertrophy, fiber disarray, increased loose connective tissue, and fibrosis, which are all thought to interfere with force generation and relaxation of the cardiac muscle. Both speckle tracking and tissue Doppler highlight this feature, with a significantly lower longitudinal systolic strain, systolic strain rate, and early diastolic strain rate (see Fig. 7) [27, 28, 30, 37]. GLS is independently associated with outcomes in HCM patients. A GLS with a value higher than –10 percent is associated with a higher risk of adverse events. This depressed contractility function of the LV correlates also with myocardial fibrosis and predicts ventricular arrhythmias. Through strain echocardiography an important prognostic parameter can be obtained: the mechanical dispersion, defined as the standard deviation of time from the onset of the QRS to peak negative strain. This is related to the amount of fibrosis and is also an independent predictor for arrhythmias [28].

## 4. Restrictive Cardiomyopathy

**Fig. 8.**
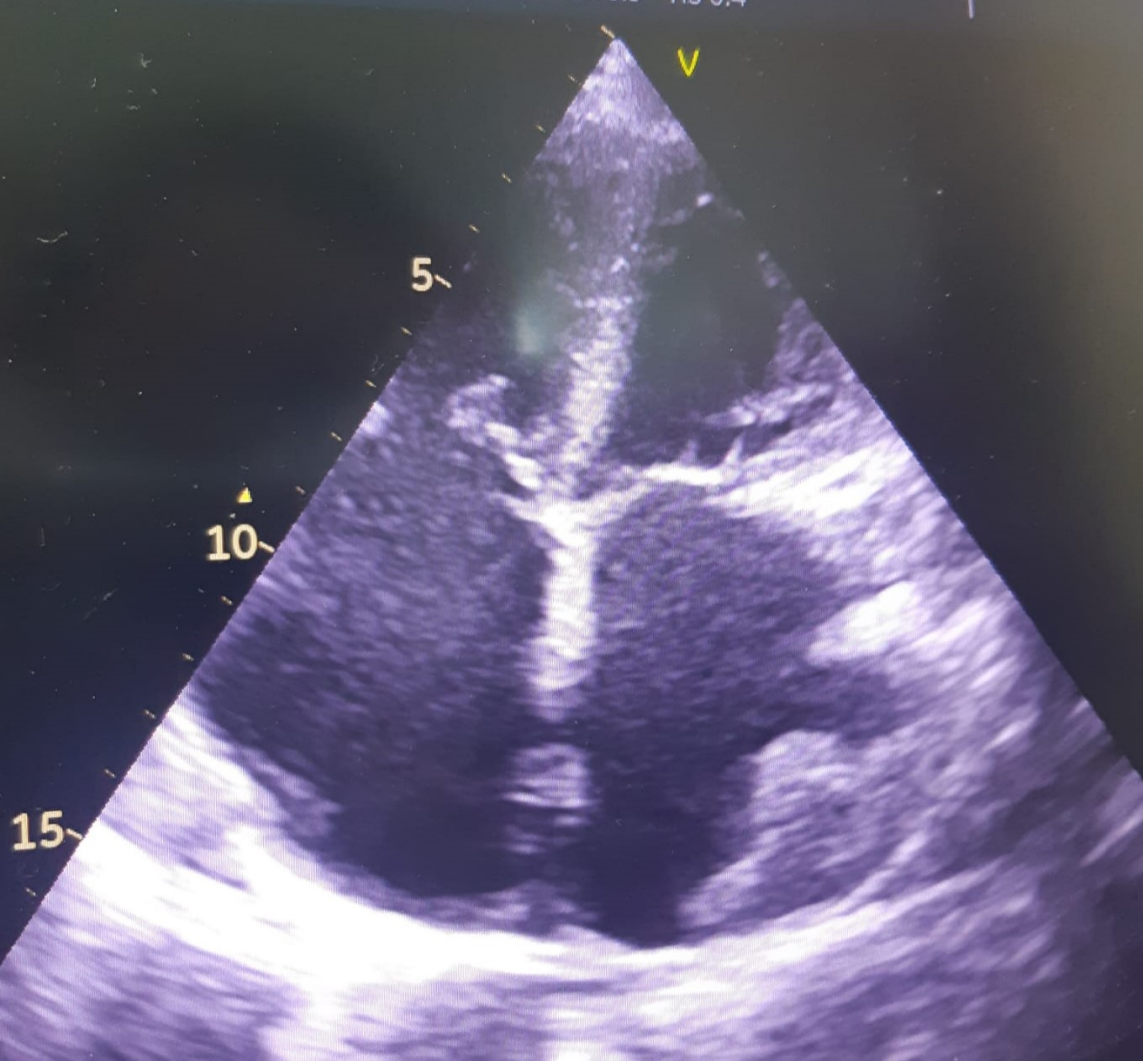
**The echocardiographic aspect of RCM in an elderly patient presenting with signs and symptoms of heart failure**. Apical 4 chamber view showing severe biatrial enlargement, with left atrial thrombus and non-dilated left and right ventricles.

Restrictive cardiomyopathy (RCM) is characterized by non-dilated ventricles, mild or no myocardial hypertrophy with impaired ventricular filling, thus it is defined by abnormal ventricular diastolic function with a normal size LV (see Fig. 8). In the early stages of the disease, the systolic function of the LV is also normal [38].

**Fig. 9.**
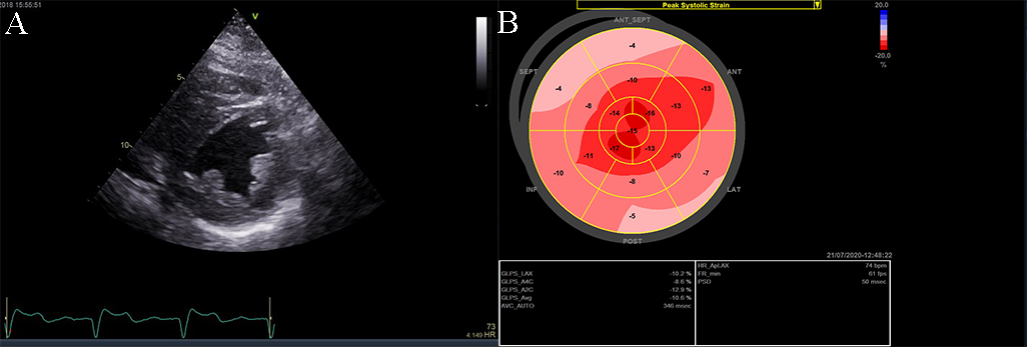
**The echocardiographic aspect of cardiac amyloidosis, associated with aortic stenosis**. (A) short-axis view, showing left ventricular hypertrophy. (B) Speckle tracking echocardiography obtained by measuring longitudinal strain in apical 4 chamber view, shows a significantly reduced GLS (global longitudinal strain) of –8.6%.

The causes of RCM can be classified as non-infiltrative (familial) or infiltrative (storage diseases), but the same pattern can be found in other disorders like diabetic cardiomyopathy, scleroderma, and endomyocardial fibrosis [38, 39, 40]. An accurate myocardial investigation by MRI and myocardial scintigraphy allows the diagnosis of different types of myocardial amyloidosis which is increasingly observed in the elderly either by transthyretin (aTTR) or by AL–light immunoglobulin chains in different plasmacytomas [41]. Another type of RCM is found in sarcoidosis and it can cause global or regional LV wall motion abnormalities. In some cases, the changes are specifically revealed on the basal posterior and lateral wall of the LV [42, 43, 44]. The most common finding is myocardial thinning, but RCM can also present with hypertrophy or it can include myocardial aneurysms [38, 45, 46, 47]. In some RCMs such as endomyocardial fibrosis or hypereosinophilic syndrome apical intraventricular thrombosis may be recognized; this may be responsible for pulmonary or systemic cardioembolism [48]. In RCM due to amyloidosis, apart from hypertrophy and wall motion abnormalities (Fig. 9), valvular involvement may also be noted with thickened aortic cusps or mitral leaflets determined by local deposition of amyloid [49].

### 4.1 2D Echocardiography

2D echocardiography, as mentioned before, highlights a low or normal diastolic volume of the LV associated with normal or only mildly reduced LV ejection fraction, as criteria for RCM. Also, one or both atrial enlargement is identified, with increased pressures, and normal pericardium. Once the typical pattern of RCM is identified, the echocardiogram can reveal also some features for a specific etiology. For example, in cardiac amyloidosis, the left and right ventricular walls are often mildly and symmetrically thickened, the myocardium may have a granular appearance, and myocardial strain imaging may show preserved apical function. However, it is not sufficient information neither to confirm, nor exclude cardiac amyloidosis [39, 40, 50], and further testing is needed. Moreover, in sarcoidosis global or regional (typically basal posterior and lateral) LV wall motion abnormalities may be observed. The most common finding is myocardial thinning, while less common findings include myocardial aneurysms, hypertrophy, and pericardial effusion [51, 52]. In eosinophilic inflammation of the myocardium or hypereosinophilic syndrome, the echocardiogram is often unrevealing during the initial necrotic stage. In the thrombotic stage of the disease, the damaged endocardium may have associated thrombus, predominantly involving the ventricular apex [3, 5, 38, 52, 53]. During the last, fibrotic stage, increased endomyocardial echogenicity is seen, affecting one or both ventricles, sometimes with overlying thrombus; the ventricular filling can be restricted, and atrioventricular valve leaflets may be tethered [53, 54, 55]. A pattern similar to hypereosinophilic syndrome can emerge in endomyocardial fibrosis. It may be characterized by LV, RV, or biventricular apical fibrosis [53, 54, 55]. In radiation-induced RCM the echocardiogram may show structural abnormalities within the field of radiation, such as calcified heart valves, a thickened pericardium, or focal wall motion abnormalities related or not to radiation-associated coronary artery disease [40, 42, 56, 57].

### 4.2 Doppler Echocardiography

**Fig. 10.**
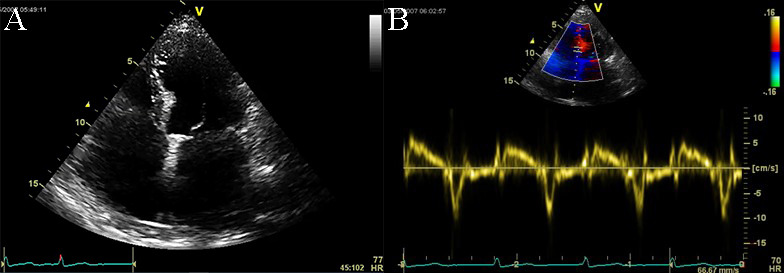
**Echocardiographic aspect of RCM**. (A) Apical 4c view of an elderly patient with idiopathic restrictive cardiomyopathy, severe biatrial enlargement (left atrium >right atrium) with atrial fibrillation. (B) PW tissue Doppler recording of the septal mitral annulus showing mildly reduced myocardial velocities (systolic velocity of 5 cm/sec). A video of the apical 4 chamber view is available online (Appendix Video 5).

Doppler echocardiography and tissue Doppler imaging show diastolic dysfunction, frequently with a restrictive pattern. Different aspects of the diastolic function can be revealed, but usually more than grade 2 diastolic dysfunction, with increased filling pressures: an elevated peak mitral inflow velocity (high velocity of E wave), rapid early mitral inflow deceleration (low deceleration time). E/A ratio greater than 0.8, deceleration time of E wave lower than 200 milliseconds, and frequently lower than 160 milliseconds, E/e’ ratio with a value above 9, are features found in the echocardiographic examination of RCM (see Fig. 10 and Appendix Video 5) [1, 4].

### 4.3 Tissue Doppler Imaging (TDI) 

TDI is a valuable method of analysis of the systolic function in patients with suspected RCM, helping in the differential diagnosis between RCM and constrictive pericarditis. It offers information related to the contraction power of regional and global LV myocardium. Reductions in TDI systolic and diastolic parameters typically occur earlier in the natural history of the amyloid disease and other types of RCM compared to other traditional echocardiographic measurements. The diastolic function in TDI assessment has restrictive pattern, with low e’, and E/e’ (average of septal and lateral mitral annulus) >13 [58, 59]. Moreover, earlier in the natural history of the restrictive disease, abnormalities of mitral annular TDI parameters may classify the patient in the mild or moderate categories of diastolic dysfunction [38, 44, 60].

### 4.4 Strain Echocardiography

Strain echocardiography reveals low myocardial velocities with regional function inhomogeneity. In RCM due to cardiac amyloidosis, both LV and right ventricle (RV) walls are often moderately, or even severely, and symmetrically thickened and the myocardium may have a granular aspect. This aspect cannot be explained by secondary causes such as hypertension. In this case, strain echocardiography may show preserved apical function [45, 46, 47]. Regarding the LA function, strain echocardiography may help in the differential diagnosis between RCM and constrictive pericarditis (CP). In both RCM and CP, the reservoir function of the left atrium is normal or may be increased compared to normal due to higher filling pressure. Contrary to this, both active and passive functions are decreased in RCM. This can be evidenced using global and regional strain, which highlights a lower value compared to normal of the septal LA strain rate [61].

## 5. Other Cardiomyopathies

### 5.1 Arrhythmogenic Cardiomyopathy

**Fig. 11.**
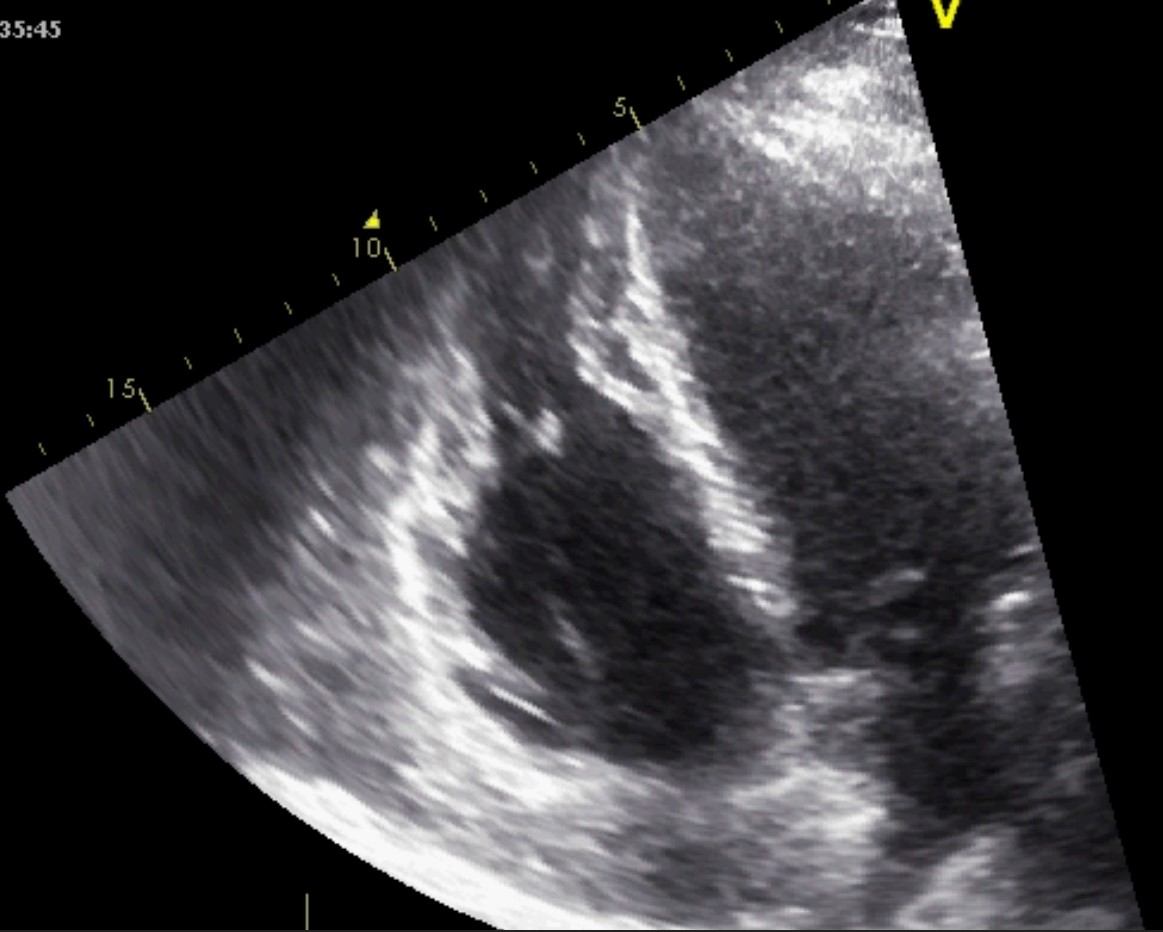
**Echocardiographic aspect of AC**. Apical 4 chamber view showing dilation and thick right ventricular free wall. A video of the apical 4 chamber view is available online (Appendix Video 6).

Arrhythmogenic cardiomyopathy (AC) is defined by fibrous and fibro-fatty replacement especially of the RV myocardium, affecting the inflow and outflow tract, but also the apex. This process most commonly affects the posterior and inferior areas of the right ventricular inflow tract adjacent to the tricuspid valve, but it also affects the anterior infundibulum and the apex, thus forming what is known as the “triangle of dysplasia”. According to recent data, there is also involvement of the postero-lateral wall of the LV. In the LV the fibro-fatty replacement determines a transmural lesion but the process starts from the subepicardial to the subendocardial LV layers. It has been demonstrated that in left ACM, the scar tissue tends to localize in the inferolateral subepicardial LV wall. In the initial assessment of AC, these typical regional wall motion abnormalities may be detected. Later changes may involve the RV free wall and become global, producing RV dilation (Fig. 11 and Appendix Video 6). Echocardiography is the first line and sometimes a sufficient method of evaluation in patients with AC.

The current guideline for a positive diagnosis involves the cardiac evaluation through echocardiography, magnetic resonance, or angiography to detect global or regional dysfunction of the RV and/or structural changes (Table 1, Ref. [62]). The right ventricle myocardial performance index (RV MPI) can be used in the baseline evaluation and follow-up for patients with AC, as it was shown to be a strong predictor for major cardiovascular adverse events, with a cut-off of 0.67.

**Table 1.** **Echocardiographic criteria for positive diagnosis—2010 revised Task Force criteria for the diagnosis of arrhythmogenic right ventricular cardiomyopathy**.

Major echocardiographic criteria	RV wall motion abnormality and 1 of the following (end diastole):
	1. PLAX RVOT ≥32 mm (corrected for body size [PLAX/BSA] ≥19 mm/$m^{2}$);
	2. PSAX RVOT ≥36 mm (corrected for body size [PSAX/BSA] ≥21 mm/$m^{2}$);
	3. or fractional area change ≤33%
Minor echocardiographic criteria	RV akinesia or dyskinesia and 1 of the following (end diastole):
	1. PLAX RVOT ≥29 to <32 mm (corrected for body size [PLAX/BSA] ≥16 to <19 mm/$m^{2}$)
	2. PSAX RVOT ≥32 to <36 mm (corrected for body size [PSAX/BSA] ≥18 to <21 mm/$m^{2}$);
	3. Or fractional area change >33% to ≤40%

Modified from Marcus *et al*., 2010 [62].

TAPSE and fractional area change, as parameters of RV systolic function assessment, are prognostic factors in AC. RV ejection fraction estimation through 3D echocardiography, lateral systolic myocardium velocities by TDI and regional systolic strain have lower values in patients with AC [62, 63, 64, 65]. However, layer-specific GLS can predict arrhythmic risk in AC [66].

### 5.2 Non-Classified Cardiomyopathies

#### 5.2.1 Ventricular Non-Compaction Cardiomyopathy.

**Fig. 12.**
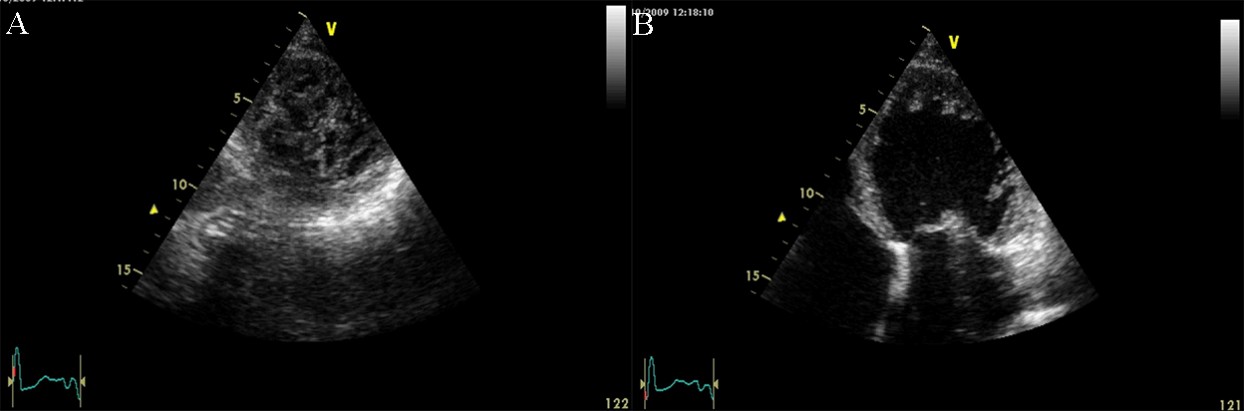
**Echocardiographic non-compaction cardiomyopathy**. (A) 2D short axis view, at the level of the apex, showing a meshwork of trabeculae. (B) 2D Apical 4 chamber view showing dilation of the left ventricle, and apical trabeculation. A video of the apical 4 chamber view is available online (Appendix Video 7).

Left ventricular non-compaction cardiomyopathy (LVNC) is characterized by an LV wall with prominent trabeculae and deep intertrabecular recesses filled with blood resulting in two layers of myocardium: a thickened noncompacted endocardial layer and an external, subepicardial thin compacted layer. The diagnosis of LVNC is obtained through morphologic criteria on transthoracic echocardiography (Fig. 12 and Appendix Video 7). The echocardiographic appearance of isolated LVNC is very heterogeneous and it can include dilated, hypertrophic or restrictive types. The Jenni criteria for echocardiographic diagnosis of LVNC are the validated imaging benchmark [67]. The necessary parameters are assessed using the parasternal short-axis view at the base, midventricular, and apical levels, and for a positive diagnosis all four of the following criteria must be met: (1) two myocardial layers: a thin compacted exterior (epicardial) and a markedly thickened endocardial layer with several prominent trabeculations and deep recesses with a maximum ratio of noncompacted to compacted myocardium greater than 2:1 at end-systole in the parasternal short-axis view; (2) evidence of flow within the deep intertrabecular recesses through color Doppler; (3) presence of prominent trabecular meshwork in the LV apex or midventricular segments of the inferior and lateral wall; (4) maximal systolic compact thickness of ≤8.1 mm. Other associated diagnosis elements are reduced global LV systolic function, diastolic dysfunction, LV thrombi, and abnormal papillary muscle structure [67, 68, 69, 70]. The absence of well-defined papillary muscles is a very typical finding of LVNC [47, 67, 68, 69, 70, 71].

#### 5.2.2 Stress Cardiomyopathy

**Fig. 13.**
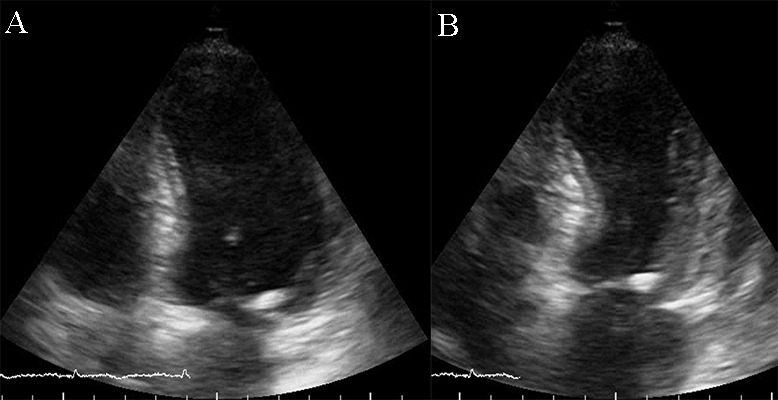
**Apical ballooning in a typical form of Takotsubo cardiomyopathy**. TTE apical 4 chamber view. (A) end diastolic frame (B) end systolic frame. A video of the apical 4 chamber view is available online (Appendix Video 8).

*Takotsubo syndrome*, initially described in 1990 by Sato *et al*. [72] in Japan, represents a sudden, usually transient, systolic cardiac dysfunction that involves LV apical akinesis and mimics an acute coronary syndrome [73]. Imaging, performed most frequently by echocardiography, shows a typical pattern of LV wall motion abnormality [74]. This includes apical dilatation with akinesia (see Fig. 13 and Appendix Video 8). There are also atypical variants including mid-ventricular, basal, focal (limited to an isolated segment), and global cases of wall contractility disorders that are stress hormone-mediated by abnormal stimulation of the cortical-hypophysis-suprarenal axis and major catecholamine release [75, 76, 77]. The standardized diagnosis uses the Mayo criteria, including clinical, biological, and imaging parameters, and all of them are necessary to complete a positive stress cardiomyopathy diagnosis [73, 78, 79, 80, 81]: (1) transient LV systolic dysfunction (hypokinesis, akinesis, or dyskinesis), apical ballooning or midventricular, basal, or focal wall motion abnormalities. Right ventricular involvement can be present; (2) the takotsubo syndrome can be preceded by an emotional, physical, or combined trigger; (3) other accepted triggers are pheochromocytoma and neurologic disorders (subarachnoid hemorrhage, stroke/transient ischemic attack, or seizures); (4) new electrocardiographic abnormalities (ST-segment elevation/depression, T-wave inversion or QTc prolongation) are usually present, but in rare occasions, no ECG changes are observed; (5) moderate elevation in cardiac troponin is common, as well as marked elevation of brain natriuretic peptide; (6) significant coronary disease may be also observed; (7) absence of the clinical context of myocarditis; (8) the most affected patient category is postmenopausal women [81]. The wall motion abnormalities are typically regional and extend beyond a single epicardial coronary artery distribution; rare exceptions are the focal (within one coronary distribution) and the global type. As described before, in the majority of cases, the regional wall abnormalities involve the apical segments of the LV, as recorded in 81.7% of the patients from the International Takotsubo Registry [80, 82, 83, 84]. The second most frequently encountered phenotype is the mid-ventricular one, with hypokinesia limited to the mid-segments of the LV, with relative sparing of the apex [78, 85]. This is present in approximately 14.6% of patients in the International Takotsubo Registry [80]. Less common phenotypes are basal, focal, or global types. Only 2.2% of patients from the International Takotsubo Registry presented basal hypokinesis with sparing of the mid-ventricle and apex, also called reverse or inverted Takotsubo (basal type) [78, 80, 85, 86]. A rare focal variant, characterized by dysfunction of an isolated segment (most commonly the anterolateral segment) of the LV, is present in 1.5% of the patients. Very rarely, in a few isolated cases, the patients have global hypokinesis [86, 87, 88]. There is a potential life-saving contribution of Speckle-tracking echocardiography (STE) for the early distinction between an acute phase of Takotsubo syndrome (TTS) and acute apical myocardial infarction, which allows avoiding the deleterious effects of catecholamine therapy in patients with Takotsubo-associated acute heart failure. Such a distinction is facilitated by the ability of STE to detect and quantify myocardial shortening (i.e., contraction) in a visually akinetic wall segment [89].

## 6. Deep Learning in Echocardiography for Diagnosis of Cardiomyopathies

Recently, automated interpretation of echocardiograms was shown to be a potentially useful method for primary care centers to obtain correct diagnoses in non-expert centers [90]. Neural network-based approaches, with deep learning models such as EchoNet, may help speed up the diagnostic process or evaluate parameters that challenging for human evaluation [91]. As this field is expanding, algorithms can be used not only for ejection fraction evaluation [92], but also for providing a differential diagnosis and etiology for patients with left ventricular hypertrophy [93]. A recent study constructed an automated algorithm that correctly discriminates between hypertensive heart disease, hypertrophic cardiomyopathy, and cardiac amyloidosis, with good sensitivity and specificity [93]. Moreover, another study showed that a machine learning algorithm was able to outperform a committee of cardiologists in discerning between Takotsubo cardiomyopathy and acute myocardial infarction [94]. This field is still in its early days, but shows promise in the diagnosis and follow-up protocols of cardiomyopathies, especially for the ones with a hypertrophic phenotype. However, for dilated cardiomyopathy, magnetic resonance imaging is preferred to echocardiography when constructing deep learning algorithms, as it provides superior spatial resolution [95, 96, 97].

## 7. Discussion

The diagnosis of every cardiomyopathy might be challenging due to the phenotype variability and the multitude of morphological aspects that might appear in each of them. The common ground in all cardiomyopathies is the presentation with heart failure signs and symptoms. Echocardiography is the first method used for evaluation, allowing an exhaustive characterization of cardiac function and morphology. Two-dimensional and Doppler echocardiography is able to define the anatomic and functional characteristics of the heart that are diagnostic of DCM, HCM, ARVC, or RCM.

To compare the different diagnostic and prognostic echocardiographic parameters presented for hypertrophic, dilated, and restrictive cardiomyopathy please refer to Table 2 (Ref. [4, 10, 11, 13, 28, 29, 30, 33, 38, 58, 59, 98, 99, 100, 101, 102, 103]).

**Table 2.** **Diagnostic and prognostic echocardiographic parameters in hypertrophic, dilated and restrictive cardiomyopathy**.

**Parameter**	**HCM**	**DCM**	**RCM**
LVEDV	N	>2 SD (>100 mL/$m^{2}$) [4, 98]	N
IVS (IVS/PWT)	>15 (>1.5) [29]	N	Depends on etiology
**Systolic function**			
RV hypertrophy	>*7 mm* [99]		
Systolic annular lateral wall velocity (S)	<4 mm [99]		
EF	N	<40–45% [98, 100]	>45%
FAC		<*27%* [101]	
GLS LV	>*-10% * [28]	*–10%* [13]	–7.5% [102]
GLS RV		*–8.6* [103]	
**Diastolic function**	*Restrictive* [33]	*Restrictive, pseudo-normal pattern* [10, 11]	Restrictive
E tricuspid flow/E’ tricuspid annulus	>*6.8* [58]		
Tricuspid EDT	*Restrictive pattern (short EDT [58])*		
LA volume	>*118 mL * [28, 30]	>50 mL/$m^{2}$ [100]	>*60 mm * [38]
LA strain	<*23.4%* [99]		
E/A mitral		>*1.6* [59]	
Mitral EDT		<*150* [59]	
E mitral		>*0.8* [59]	
E mitral flow/E’ mitral annulus	>*14* [103]		
E’	<*9* [103]		
Left Ventricular Relaxation Half-Time	>*38.5 msec* [103]	>*41 msec* [103]	

EDT, E wave deceleration time; EF, ejection fraction; FAC, fractional area change; GLS, global longitudinal strain; IVS, interventricular septum; LA, Left atrium; LVEDV, left ventricle end,diastolic volume; N,normal dimension; PWT, posterior wall thickness; RV, right ventricle; SD, standard deviation. In italics, parameters with prognostic values, in normal font, parameters with diagnostic values.

The systolic function of the LV and RV is established through a variety of echocardiographic parameters, the quantitative bidimensional LV ejection fraction can be easily performed in medical facilities all over the world. When systolic dysfunction occurs, cardiac output is initially maintained with two consequences: left atrium enlargement and increased contractility through the Frank-Starling mechanism. However, these compensatory mechanisms are eventually exceeded, and cardiac output decreases, resulting in clinical manifestations of heart failure. Systolic dysfunction is a main feature of DCM. It is also seen in some patients with HCM who develop progressive LV wall thinning and a small increase in its diastolic dimensions. Relative wall thickness (RWT) is defined as two times posterior wall thickness divided by the LV diastolic diameter, is a measure of LV geometry and can be a marker for adverse events in patients with LV systolic dysfunction. Concentric LV hypertrophy (RWT >0.42) is associated with a higher risk of stroke [37]. Tissue Doppler imaging and speckle tracking echocardiography techniques can provide more sensitive and specific information about myocardial contractile function including myocardial velocities, strain, and strain rate. These methods allow assessment of the various components of contraction, including radial, longitudinal, and circumferential contraction, allowing for assessment of global and regional systolic function [104, 105, 106, 107]. Regional evaluation of LV function is commonly determined based on qualitative visual assessment of wall thickening and endocardial motion of each myocardial segment visualized in multiple views, but parameters such as global longitudinal strain or myocardial velocities, are more sensitive for detection of decreased LV function than LV ejection fraction and is permanently recommended whenever possible as complementary clinical techniques that offer incremental prognostic information, as is the case with most cardiomyopathies. Moreover, tissue Doppler imaging and speckle tracking are important not only for a positive diagnosis of LV dysfunction, but also through the related parameters, that have a role in establishing a differential diagnosis between cardiomyopathies. HCM and RCM have normal LVEF for many years during the progression of the disease. However, myocardial cells have arrangement disarray with discretely impaired systolic and diastolic function as seen in HCM or infiltration of heart tissue with poor contractility as seen in different etiologies of RCM.

Diastolic dysfunction can be present with or without associated systolic dysfunction. When systolic dysfunction is described, diastolic dysfunction is always present [4, 108, 109]. Otherwise, in patients with heart failure and normal systolic function, diastolic dysfunction is one of the potential causes. Causes of HF with a normal or near-normal LVEF include many cardiomyopathies with preserved ejection fraction (e.g., HCM, RCM, LV non-compaction) [108, 110]. The LV diastole includes two phases: the first is the relaxation phase, which is a dynamic process that takes place during isovolumic relaxation and the second phase happens during early rapid filling of the ventricle [108, 111]. Later in diastole, after relaxation is complete, further LV filling is a passive process that is dependent on the compliance or distensibility of the myocardium and ends in the atrial active filling phase. Either active relaxation or passive compliance or both may be impaired in a patient with diastolic dysfunction. Echocardiographic evaluation of LV diastolic dysfunction includes Doppler assessment of transmitral flow and pulmonary venous flow, as well as tissue Doppler imaging, which is characteristic of both HCM and RCM [112, 113]. LV dimensions, left atrium maximum volume, LV wall thickness, volumes, segmental function, global longitudinal strain, and LV are parameters needed to assess the diastolic dysfunction. Indices of diastolic function include mitral inflow velocities, mitral annular velocities by tissue Doppler (septal and lateral), and pulmonary vein velocities. Isovolumic relaxation time (IVRT), tricuspid inflow velocities, and color Doppler early diastolic flow propagation velocity (Vp). In addition, the latest recommendation is the assessment of left atrium strain, particularly in cases with incomplete/suboptimal Doppler signals or indeterminate diastolic function [114, 115].

## 8. Conclusions and Future Directions

Echocardiography is recognized as a first-line diagnostic method in almost all cardiac diseases. When dealing with a patient with cardiomyopathy, echocardiography is a mandatory tool for both positive or differential diagnostic and follow-up. Although some cases cannot be completely characterized without the aid of genetic testing, MRI, etc., it is essential to have a working diagnosis after the first echocardiographic examination, as this will establish all future testing. Traditional and also newer echocardiographic techniques, that identify diagnostic and prognostic parameters, are the cornerstone in the management of cardiomyopathies. The ability of modern techniques to identify subclinical disease plays an important role in the long-term evolution of cardiomyopathy patients. Machine learning algorithms are the future in the assessment of several pathologies. Related to the echocardiographic evaluation of cardiomyopathies, it is possible to obtain machine algorithms to establish a differential diagnosis and prognostic patterns. However, for the time being, the accuracy of the obtained information is improved when associated with other clinical and paraclinical methods.

## References

[1] Wood MJ (2004). Utility of echocardiography in the evaluation of individuals with cardiomyopathy. *Heart*.

[2] Elliott PM (2013). Classification of Cardiomyopathies: Evolution or revolution. *Journal of the American College of Cardiology*.

[3] Maron BJ, Towbin JA, Thiene G, Antzelevitch C, Corrado D, Arnett D (2006). Contemporary definitions and classification of the cardiomyopathies: An American Heart Association Scientific Statement from the Council on Clinical Cardiology, Heart Failure and Transplantation Committee; Quality of Care and Outcomes Research and Functio. *Circulation*.

[4] Lang RM, Badano LP, Mor-Avi V, Afilalo J, Armstrong A, Ernande L (2015). Recommendations for Cardiac Chamber Quantification by Echocardiography in Adults: an Update from the American Society of Echocardiography and the European Association of Cardiovascular Imaging. *Journal of the American Society of Echocardiography*.

[5] Agler DA, Adams DB, Waggoner AD (2007). Cardiac Resynchronization Therapy and the Emerging Role of Echocardiography (Part 2): the Comprehensive Examination. *Journal of the American Society of Echocardiography*.

[6] Park MY (2020). Prognostic Implications of Left Ventricular Global Longitudinal Strain in Dilated Cardiomyopathy. *Journal of Cardiovascular Imaging*.

[7] Redfield MM, Jacobsen SJ, Burnett JC, Mahoney DW, Bailey KR, Rodeheffer RJ (2003). Burden of Systolic and Diastolic Ventricular Dysfunction in the Community: appreciating the scope of the heart failure epidemic. *The Journal of the American Medical Association*.

[8] Mele D, Pasanisi G, Capasso F, De Simone A, Morales M, Poggio D (2006). Left intraventricular myocardial deformation dyssynchrony identifies responders to cardiac resynchronization therapy in patients with heart failure. *European Heart Journal*.

[9] Hoit BD (2014). Left Atrial Size and Function: role in prognosis. *Journal of the American College of Cardiology*.

[10] Ristow B, Na B, Ali S, Whooley MA, Schiller NB (2011). Left Ventricular Outflow Tract and Pulmonary Artery Stroke Distances Independently Predict Heart Failure Hospitalization and Mortality: the Heart and Soul Study. *Journal of the American Society of Echocardiography*.

[11] Ghio S, Recusani F, Klersy C, Sebastiani R, Laudisa ML, Campana C (2000). Prognostic usefulness of the tricuspid annular plane systolic excursion in patients with congestive heart failure secondary to idiopathic or ischemic dilated cardiomyopathy. *The American Journal of Cardiology*.

[12] Chahal NS, Lim TK, Jain P, Chambers JC, Kooner JS, Senior R (2010). Normative reference values for the tissue Doppler imaging parameters of left ventricular function: a population-based study. *European Journal of Echocardiography*.

[13] Jung IH, Park JH, Lee J, Kim GS, Lee HY, Byun YS (2020). Left Ventricular Global Longitudinal Strain as a Predictor for Left Ventricular Reverse Remodeling in Dilated Cardiomyopathy. *Journal of Cardiovascular Imaging*.

[14] Yu C, Sanderson JE, Gorcsan J (2010). Echocardiography, dyssynchrony, and the response to cardiac resynchronization therapy. *European Heart Journal*.

[15] Marcus GM, Rose E, Viloria EM, Schafer J, De Marco T, Saxon LA (2005). Septal to Posterior Wall Motion Delay Fails to Predict Reverse Remodeling or Clinical Improvement in Patients Undergoing Cardiac Resynchronization Therapy. *Journal of the American College of Cardiology*.

[16] McMurray JJ, Adamopoulos S, Anker SD, Auricchio A, Böhm M, Dickstein K (2012). ESC Guidelines for the diagnosis and treatment of acute and chronic heart failure 2012: The Task Force for the Diagnosis and Treatment of Acute and Chronic Heart Failure 2012 of the European Society of Cardiology. Developed in collaboration with the Heart Failure Association (HFA) of the ESC. *European Heart Journal*.

[17] Parsai C, Bijnens B, Sutherland GR, Baltabaeva A, Claus P, Marciniak M (2009). Toward understanding response to cardiac resynchronization therapy: left ventricular dyssynchrony is only one of multiple mechanisms. *European Heart Journal*.

[18] Glikson M, Nielsen JC, Kronborg MB, Michowitz Y, Auricchio A, Barbash IM (2021). 2021 ESC Guidelines on cardiac pacing and cardiac resynchronization therapy. *European Heart Journal*.

[19] Penicka M, Bartunek J, De Bruyne B, Vanderheyden M, Goethals M, De Zutter M (2004). Improvement of Left Ventricular Function after Cardiac Resynchronization Therapy is Predicted by Tissue Doppler Imaging Echocardiography. *Circulation*.

[20] Bax JJ, Marwick TH, Molhoek SG, Bleeker GB, van Erven L, Boersma E (2003). Left ventricular dyssynchrony predicts benefit of cardiac resynchronization therapy in patients with end-stage heart failure before pacemaker implantation. *The American Journal of Cardiology*.

[21] Chung ES, Leon AR, Tavazzi L, Sun J, Nihoyannopoulos P, Merlino J (2008). Results of the Predictors of Response to CRT (PROSPECT) Trial. *Circulation*.

[22] Priori SG, Blomström-Lundqvist C, Mazzanti A, Blom N, Borggrefe M, Camm J (2015). 015 ESC Guidelines for the management of patients with ventricular arrhythmias and the prevention of sudden cardiac death the Task Force for the Management of Patients with Ventricular Arrhythmias and the Prevention of Sudden Cardiac Death of the Europea. *European Heart Journal*.

[23] Ommen SR, Mital S, Burke MA, Day SM, Deswal A, Elliott P (2020). 2020 AHA/ACC Guideline for the Diagnosis and Treatment of Patients with Hypertrophic Cardiomyopathy: Executive Summary: A Report of the American College of Cardiology/American Heart Association Joint Committee on Clinical Practice Guidelines. *Journal of the American College of Cardiology*.

[24] Neubauer S, Kolm P, Ho CY, Kwong RY, Desai MY, Dolman SF (2019). Distinct Subgroups in Hypertrophic Cardiomyopathy in the NHLBI HCM Registry. *Journal of the American College of Cardiology*.

[25] Dimitrow PP, Rajtar-Salwa R (2020). Reversed Septal Curvature Predicts Sudden Death in Hypertrophic Cardiomyopathy in Earlier Study. *Journal of the American College of Cardiology*.

[26] Dimitrow PP (2005). Echocardiographic risk factors predisposing to sudden cardiac death in hypertrophic cardiomyopathy. *Heart*.

[27] Panza JA, Petrone RK, Fananapazir L, Maron BJ (1992). Utility of continuous wave doppler echocardiography in the noninvasive assessment of left ventricular outflow tract pressure gradient in patients with hypertrophic cardiomyopathy. *Journal of the American College of Cardiology*.

[28] Haland TF, Almaas VM, Hasselberg NE, Saberniak J, Leren IS, Hopp E (2016). Strain echocardiography is related to fibrosis and ventricular arrhythmias in hypertrophic cardiomyopathy. *European Heart Journal: Cardiovascular Imaging*.

[29] Gersh BJ, Maron BJ, Bonow RO, Dearani JA, Fifer MA, Link MS (2011). 2011 ACCF/AHA Guideline for the Diagnosis and Treatment of Hypertrophic Cardiomyopathy: executive summary: a report of the American College of Cardiology Foundation/American Heart Association Task Force on Practice Guidelines. *Circulation*.

[30] Maron BJ, Gottdiener JS, Arce J, Rosing DR, Wesley YE, Epstein SE (1985). Dynamic subaortic obstruction in hypertrophic cardiomyopathy: Analysis by pulsed doppler echocardiography. *Journal of the American College of Cardiology*.

[31] Rowin EJ, Maron BJ, Haas TS, Garberich RF, Wang W, Link MS (2017). Hypertrophic Cardiomyopathy with Left Ventricular Apical Aneurysm: Implications for Risk Stratification and Management. *Journal of the American College of Cardiology*.

[32] Papanastasiou CA, Zegkos T, Karamitsos TD, Rowin EJ, Maron MS, Parcharidou D (2021). Prognostic role of left ventricular apical aneurysm in hypertrophic cardiomyopathy: A systematic review and meta-analysis. *International Journal of Cardiology*.

[33] Zamorano JL, Anastasakis A, Borger MA, Borggrefe M, Cecchi F, Charron P (2014). 2014 ESC guidelines on diagnosis and management of hypertrophic cardiomyopathy: The task force for the diagnosis and management of hypertrophic cardiomyopathy of the European Society of Cardiology (ESC). *European Heart Journal*.

[34] Dandel M, Hetzer R (2021). Ventricular systolic dysfunction with and without altered myocardial contractility: Clinical value of echocardiography for diagnosis and therapeutic decision-making. *International Journal of Cardiology*.

[35] Schannwell CM, Zimmermann T, Schneppenheim M, Plehn G, Marx R, Strauer BE (2002). Left Ventricular Hypertrophy and Diastolic Dysfunction in Healthy Pregnant Women. *Cardiology*.

[36] Nagueh SF, McFalls J, Meyer D, Hill R, Zoghbi WA, Tam JW (2003). Tissue Doppler Imaging Predicts the Development of Hypertrophic Cardiomyopathy in Subjects With Subclinical Disease. *Circulation*.

[37] Klues HG, Roberts WC, Maron BJ (1993). Morphological determinants of echocardiographic patterns of mitral valve systolic anterior motion in obstructive hypertrophic cardiomyopathy. *Circulation*.

[38] Ammash NM, Seward JB, Bailey KR, Edwards WD, Tajik AJ (2000). Clinical Profile and Outcome of Idiopathic Restrictive Cardiomyopathy. *Circulation*.

[39] Gilstrap LG, Dominici F, Wang Y, El-Sady MS, Singh A, Di Carli MF (2019). Epidemiology of Cardiac Amyloidosis-Associated Heart Failure Hospitalizations among Fee-for-Service Medicare Beneficiaries in the United States. *Circulation: Heart Failure*.

[40] McKenna WJ, Maron BJ, Thiene G (2017). Classification, Epidemiology, and Global Burden of Cardiomyopathies. *Circulation Research*.

[41] Oda S, Kidoh M, Nagayama Y, Takashio S, Usuku H, Ueda M (2020). Trends in Diagnostic Imaging of Cardiac Amyloidosis: Emerging Knowledge and Concepts. *RadioGraphics*.

[42] Cahill TJ, Ashrafian H, Watkins H (2013). Genetic Cardiomyopathies Causing Heart Failure. *Circulation Research*.

[43] DePasquale EC, Nasir K, Jacoby DL (2012). Outcomes of adults with restrictive cardiomyopathy after heart transplantation. *The Journal of Heart and Lung Transplantation*.

[44] Grupper A, Park SJ, Pereira NL, Schettle SD, Gerber Y, Topilsky Y (2015). Role of ventricular assist therapy for patients with heart failure and restrictive physiology: Improving outcomes for a lethal disease. *The Journal of Heart and Lung Transplantation*.

[45] Cyrille NB, Goldsmith J, Alvarez J, Maurer MS (2014). Prevalence and Prognostic Significance of Low QRS Voltage among the Three Main Types of Cardiac Amyloidosis. *The American Journal of Cardiology*.

[46] Mussinelli R, Salinaro F, Alogna A, Boldrini M, Raimondi A, Musca F (2013). Diagnostic and Prognostic Value of Low QRS Voltages in Cardiac AL Amyloidosis. *Annals of Noninvasive Electrocardiology*.

[47] Elliott P, Andersson B, Arbustini E, Bilinska Z, Cecchi F, Charron P (2008). Classification of the cardiomyopathies: a position statement from the European Society Of Cardiology Working Group on Myocardial and Pericardial Diseases. *European Heart Journal*.

[48] Kariyanna PT, Hossain NA, Onkaramurthy NJ, Jayarangaiah A, Hossain NA, Jayarangaiah A (2021). Hypereosinophilia and Löffler’s Endocarditis: A Systematic Review. *American Journal of Medical Case Reports*.

[49] Rosenblum H, Masri A, Narotsky DL, Goldsmith J, Hamid N, Hahn RT (2021). Unveiling outcomes in coexisting severe aortic stenosis and transthyretin cardiac amyloidosis. *European Journal of Heart Failure*.

[50] Bellavia D, Pellikka PA, Abraham TP, Al-Zahrani GB, Dispenzieri A, Oh JK (2008). Evidence of Impaired Left Ventricular Systolic Function by Doppler Myocardial Imaging in Patients with Systemic Amyloidosis and no Evidence of Cardiac Involvement by Standard Two-Dimensional and Doppler Echocardiography. *The American Journal of Cardiology*.

[51] Lam CSP, Tolep KA, Metke MP, Glockner J, Cooper LT (2009). Coronary Sarcoidosis Presenting as Acute Coronary Syndrome. *Clinical Cardiology*.

[52] Chen W, Jeudy J (2019). Assessment of Myocarditis: Cardiac MR, PET/CT, or PET/MR. *Current Cardiology Reports*.

[53] Crane MM, Chang CM, Kobayashi MG, Weller PF (2010). Incidence of myeloproliferative hypereosinophilic syndrome in the United States and an estimate of all hypereosinophilic syndrome incidence. *Journal of Allergy and Clinical Immunology*.

[54] Weller P, Bubley G (1994). The idiopathic hypereosinophilic syndrome. *Blood*.

[55] Ogbogu PU, Bochner BS, Butterfield JH, Gleich GJ, Huss-Marp J, Kahn JE (2009). Hypereosinophilic syndrome: a multicenter, retrospective analysis of clinical characteristics and response to therapy. *Journal of Allergy and Clinical Immunology*.

[56] Pereira NL, Grogan M, Dec GW (2018). Spectrum of Restrictive and Infiltrative Cardiomyopathies: Part 1 of a 2-Part Series. *Journal of the American College of Cardiology*.

[57] Ruberg FL, Grogan M, Hanna M, Kelly JW, Maurer MS (2019). Transthyretin Amyloid Cardiomyopathy: JACC State-of-the-Art Review. *Journal of the American College of Cardiology*.

[58] Pagourelias ED, Efthimiadis GK, Parcharidou DG, Gossios TD, Kamperidis V, Karoulas T (2011). Prognostic value of right ventricular diastolic function indices in hypertrophic cardiomyopathy. *European Journal of Echocardiography*.

[59] Werner GS, Fuchs JB, Schulz R, Figulla HR, Kreuzer H (1996). Changes in left ventricular filling during follow-up study in survivors and nonsurvivors of idiopathic dilated cardiomyopathy. *Journal of Cardiac Failure*.

[60] Ditaranto R, Caponetti AG, Ferrara V, Parisi V, Minnucci M, Chiti C (2022). Pediatric Restrictive Cardiomyopathies. *Frontiers in Pediatrics*.

[61] Liu S, Ma C, Ren W, Zhang J, Li N, Yang J (2015). Regional left atrial function differentiation in patients with constrictive pericarditis and restrictive cardiomyopathy: a study using speckle tracking echocardiography. *The International Journal of Cardiovascular Imaging*.

[62] Marcus FI, McKenna WJ, Sherrill D, Basso C, Bauce B, Bluemke DA (2010). Diagnosis of arrhythmogenic right ventricular cardiomyopathy/dysplasia: Proposed Modification of the Task Force Criteria. *European Heart Journal*.

[63] Saguner AM, Vecchiati A, Baldinger SH, Rüeger S, Medeiros-Domingo A, Mueller-Burri AS (2014). Different Prognostic Value of Functional Right Ventricular Parameters in Arrhythmogenic Right Ventricular Cardiomyopathy/Dysplasia. *Circulation: Cardiovascular Imaging*.

[64] Kjaergaard J, Hastrup Svendsen J, Sogaard P, Chen X, Bay Nielsen H, Køber L (2007). Advanced Quantitative Echocardiography in Arrhythmogenic Right Ventricular Cardiomyopathy. *Journal of the American Society of Echocardiography*.

[65] Réant P, Hauer AD, Castelletti S, Pantazis A, Rosmini S, Cheang MH (2016). Epicardial myocardial strain abnormalities may identify the earliest stages of arrhythmogenic cardiomyopathy. *The International Journal of Cardiovascular Imaging*.

[66] Segura-Rodríguez D, Bermúdez-Jiménez FJ, González-Camacho L, Moreno Escobar E, García-Orta R, Alcalá-López JE (2021). Layer-Specific Global Longitudinal Strain Predicts Arrhythmic Risk in Arrhythmogenic Cardiomyopathy. *Frontiers in Cardiovascular Medicine*.

[67] Jenni R, Oechslin E, Schneider J, Attenhofer Jost C, Kaufmann PA (2001). Echocardiographic and pathoanatomical characteristics of isolated left ventricular non-compaction: a step towards classification as a distinct cardiomyopathy. *Heart*.

[68] Hershberger RE, Morales A, Cowan J (2017). Is Left Ventricular Noncompaction a Trait, Phenotype, or Disease? The Evidence Points to Phenotype. *Circulation: Cardiovascular Genetics*.

[69] Caselli S, Attenhofer Jost CH, Jenni R, Pelliccia A (2015). Left Ventricular Noncompaction Diagnosis and Management Relevant to Pre-participation Screening of Athletes. *The American Journal of Cardiology*.

[70] Lowery MH, Martel JA, Zambrano JP, Ferreira A, Eco L, Gallagher A (2003). Noncompaction of the ventricular myocardium: the use of contrast-enhanced echocardiography in diagnosis. *Journal of the American Society of Echocardiography*.

[71] Gebhard C, Stähli BE, Greutmann M, Biaggi P, Jenni R, Tanner FC (2012). Reduced Left Ventricular Compacta Thickness: a Novel Echocardiographic Criterion for Non-Compaction Cardiomyopathy. *Journal of the American Society of Echocardiography*.

[72] Dote K, Sato H, Tateishi H, Uchida T, Ishihara M (1991). Myocardial stunning due to simultaneous multivessel coronary spasms: a review of 5 cases. *Journal of Cardiology*.

[73] Bybee KA, Kara T, Prasad A, Lerman A, Barsness GW, Wright RS (2004). Systematic Review: Transient Left Ventricular Apical Ballooning: a Syndrome that Mimics ST-Segment Elevation Myocardial Infarction. *Annals of Internal Medicine*.

[74] Sato H, Tateishi H, Uchida T, Dote K, Ishihara M, Kodama K (1990). Clinical aspect of myocardial injury: from ischemia to heart failure. *Kagaku Hyoronsha*.

[75] Abe Y, Kondo M, Matsuoka R, Araki M, Dohyama K, Tanio H (2003). Assessment of clinical features in transient left ventricular apical ballooning. *Journal of the American College of Cardiology*.

[76] Sharkey SW, Lesser JR, Zenovich AG, Maron MS, Lindberg J, Longe TF (2005). Acute and Reversible Cardiomyopathy Provoked by Stress in Women from the United States. *Circulation*.

[77] Akashi YJ, Goldstein DS, Barbaro G, Ueyama T (2008). Takotsubo Cardiomyopathy: A New Form of Acute, Reversible Heart Failure. *Circulation*.

[78] Eitel I, von Knobelsdorff-Brenkenhoff F, Bernhardt P, Carbone I, Muellerleile K, Aldrovandi A (2011). Clinical Characteristics and Cardiovascular Magnetic Resonance Findings in Stress (Takotsubo) Cardiomyopathy. *The Journal of the American Medical Association*.

[79] Prasad A, Lerman A, Rihal CS (2008). Apical ballooning syndrome (Tako-Tsubo or stress cardiomyopathy): a mimic of acute myocardial infarction. *American Heart Journal*.

[80] Templin C, Ghadri JR, Diekmann J, Napp LC, Bataiosu DR, Jaguszewski M (2015). Clinical Features and Outcomes of Takotsubo (Stress) Cardiomyopathy. *New England Journal of Medicine*.

[81] Ghadri J, Wittstein IS, Prasad A, Sharkey S, Dote K, Akashi YJ (2018). International Expert Consensus Document on Takotsubo Syndrome (Part I): Clinical Characteristics, Diagnostic Criteria, and Pathophysiology. *European Heart Journal*.

[82] Tsuchihashi K, Ueshima K, Uchida T, Oh-mura N, Kimura K, Owa M (2001). Transient left ventricular apical ballooning without coronary artery stenosis: a novel heart syndrome mimicking acute myocardial infarction. Angina Pectoris-Myocardial Infarction Investigations in Japan. *Journal of the American College of Cardiology*.

[83] Ghadri JR, Cammann VL, Templin C (2016). The International Takotsubo Registry: Rationale, Design, Objectives, and First Results. *Heart Failure Clinics*.

[84] Ghadri JR, Templin C (2016). The InterTAK Registry for Takotsubo Syndrome. *European Heart Journal*.

[85] Kurowski V, Kaiser A, von Hof K, Killermann DP, Mayer B, Hartmann F (2007). Apical and Midventricular Transient Left Ventricular Dysfunction Syndrome (Tako-Tsubo Cardiomyopathy) Frequency, Mechanisms, and Prognosis. *Chest*.

[86] Win CM, Pathak A, Guglin M (2011). Not Takotsubo: a Different Form of Stress-Induced Cardiomyopathy-a Case Series. *Congestive Heart Failure*.

[87] Hussain MA, Cox AT, Bastiaenen R, Prasad A (2017). Apical ballooning (takotsubo) syndrome with concurrent ST-segment elevation myocardial infarction. *BMJ Case Reports*.

[88] Hurtado Rendón IS, Alcivar D, Rodriguez-Escudero JP, Silver K (2018). Acute Myocardial Infarction and Stress Cardiomyopathy are not Mutually Exclusive. *The American Journal of Medicine*.

[89] Dandel M, Hetzer R (2018). Deleterious effects of catecholamine administration in acute heart failure caused by unrecognized Takotsubo cardiomyopathy. *BMC Cardiovascular Disorders*.

[90] Zhang J, Gajjala S, Agrawal P, Tison GH, Hallock LA, Beussink-Nelson L (2018). Fully Automated Echocardiogram Interpretation in Clinical Practice. *Circulation*.

[91] Ghorbani A, Ouyang D, Abid A, He B, Chen JH, Harrington RA (2020). Deep learning interpretation of echocardiograms. *Npj Digital Medicine*.

[92] Liu X, Fan Y, Li S, Chen M, Li M, Hau WK (2021). Deep learning-based automated left ventricular ejection fraction assessment using 2-D echocardiography. *American Journal of Physiology-Heart and Circulatory Physiology*.

[93] Yu X, Yao X, Wu B, Zhou H, Xia S, Su W (2022). Using deep learning method to identify left ventricular hypertrophy on echocardiography. *The International Journal of Cardiovascular Imaging*.

[94] Laumer F, Di Vece D, Cammann VL, Würdinger M, Petkova V, Schönberger M (2022). Assessment of Artificial Intelligence in Echocardiography Diagnostics in Differentiating Takotsubo Syndrome from Myocardial Infarction. *JAMA Cardiology*.

[95] Miller R, Kerfoot E, Mauger C, Ismail TF, Young AA, Nordsletten DA (2021). An Implementation of Patient-Specific Biventricular Mechanics Simulations With a Deep Learning and Computational Pipeline. *Frontiers in Physiology*.

[96] MacGregor RM, Guo A, Masood MF, Cupps BP, Ewald GA, Pasque MK (2021). Machine Learning Outcome Prediction in Dilated Cardiomyopathy Using Regional Left Ventricular Multiparametric Strain. *Annals of Biomedical Engineering*.

[97] Asher C, Puyol-Antón E, Rizvi M, Ruijsink B, Chiribiri A, Razavi R (2021). The Role of AI in Characterizing the DCM Phenotype. *Frontiers in Cardiovascular Medicine*.

[98] Pinto YM, Elliott PM, Arbustini E, Adler Y, Anastasakis A, Böhm M (2016). Proposal for a revised definition of dilated cardiomyopathy, hypokinetic non-dilated cardiomyopathy, and its implications for clinical practice: a position statement of the ESC working group on myocardial and pericardial diseases. *European Heart Journal*.

[99] Mandeş L, Roşca M, Ciupercă D, Popescu BA (2020). The role of echocardiography for diagnosis and prognostic stratification in hypertrophic cardiomyopathy. *Journal of Echocardiography*.

[100] Charron P, Elliott PM, Gimeno JR, Caforio ALP, Kaski JP, Tavazzi L (2018). The Cardiomyopathy Registry of the EURObservational Research Programme of the European Society of Cardiology: Baseline data and contemporary management of adult patients with cardiomyopathies. *European Heart Journal*.

[101] Ishiwata J, Daimon M, Nakanishi K, Sugimoto T, Kawata T, Shinozaki T (2021). Combined evaluation of right ventricular function using echocardiography in non‐ischaemic dilated cardiomyopathy. *ESC Heart Failure*.

[102] Bellavia D, Pellikka PA, Al-Zahrani GB, Abraham TP, Dispenzieri A, Miyazaki C (2010). Independent Predictors of Survival in Primary Systemic (AL) Amyloidosis, Including Cardiac Biomarkers and Left Ventricular Strain Imaging: an Observational Cohort Study. *Journal of the American Society of Echocardiography*.

[103] Yokoi T, Morimoto R, Oishi H, Kato H, Arao Y, Yamaguchi S (2019). Left Ventricular Relaxation Half-Time as a Predictor of Cardiac Events in Idiopathic Dilated Cardiomyopathy and Hypertrophic Cardiomyopathy with Left Ventricular Systolic and/or Diastolic Dysfunction. *The American Journal of Cardiology*.

[104] Oikonomou EK, Kokkinidis DG, Kampaktsis PN, Amir EA, Marwick TH, Gupta D (2019). Assessment of Prognostic Value of Left Ventricular Global Longitudinal Strain for Early Prediction of Chemotherapy-Induced Cardiotoxicity: A Systematic Review and Meta-analysis. *JAMA Cardiology*.

[105] Adamo L, Perry A, Novak E, Makan M, Lindman BR, Mann DL (2017). Abnormal Global Longitudinal Strain Predicts Future Deterioration of Left Ventricular Function in Heart Failure Patients with a Recovered Left Ventricular Ejection Fraction. *Circulation: Heart Failure*.

[106] Bijvoet GP, Teske AJ, Chamuleau SAJ, Hart EA, Jansen R, Schaap J (2020). Global longitudinal strain to predict left ventricular dysfunction in asymptomatic patients with severe mitral valve regurgitation: literature review. *Netherlands Heart Journal*.

[107] Potter E, Marwick TH (2018). Assessment of Left Ventricular Function by Echocardiography: The Case for Routinely Adding Global Longitudinal Strain to Ejection Fraction. *JACC: Cardiovascular Imaging*.

[108] Nagueh SF, Smiseth OA, Appleton CP, Byrd BF, Dokainish H, Edvardsen T (2016). Recommendations for the Evaluation of Left Ventricular Diastolic Function by Echocardiography: an Update from the American Society of Echocardiography and the European Association of Cardiovascular Imaging. *Journal of the American Society of Echocardiography*.

[109] Lang RM, Badano LP, Mor-Avi V, Afilalo J, Armstrong A, Ernande L (2015). Recommendations for Cardiac Chamber Quantification by Echocardiography in Adults: an Update from the American Society of Echocardiography and the European Association of Cardiovascular Imaging. *European Heart Journal: Cardiovascular Imaging*.

[110] Quiñones MA, Otto CM, Stoddard M, Waggoner A, Zoghbi WA (2002). Recommendations for quantification of Doppler echocardiography: a report from the Doppler quantification task force of the nomenclature and standards committee of the American Society of Echocardiography. *Journal of the American Society of Echocardiography*.

[111] Schwammenthal E, Popescu BA, Popescu AC, Di Segni E, Guetta V, Rath S (2004). Association of left ventricular filling parameters assessed by pulsed wave Doppler and color M-mode Doppler echocardiography with left ventricular pathology, pulmonary congestion, and left ventricular end-diastolic pressure. *The American Journal of Cardiology*.

[112] Inoue K, Khan FH, Remme EW, Ohte N, García-Izquierdo E, Chetrit M (2021). Determinants of left atrial reservoir and pump strain and use of atrial strain for evaluation of left ventricular filling pressure. *European Heart Journal - Cardiovascular Imaging*.

[113] Rivas-Gotz C, Manolios M, Thohan V, Nagueh SF (2003). Impact of left ventricular ejection fraction on estimation of left ventricular filling pressures using tissue Doppler and flow propagation velocity. *The American Journal of Cardiology*.

[114] Voigt J, Mălăescu G, Haugaa K, Badano L (2020). How to do LA strain. *European Heart Journal - Cardiovascular Imaging*.

[115] Badano LP, Kolias TJ, Muraru D, Abraham TP, Aurigemma G, Edvardsen T (2018). Standardization of left atrial, right ventricular, and right atrial deformation imaging using two-dimensional speckle tracking echocardiography: a consensus document of the EACVI/ASE/Industry Task Force to standardize deformation imaging. *European Heart Journal: Cardiovascular Imaging*.

